# Treatment-specific interrupted time series analyses of judicial deference to health technology assessment in Brazil

**DOI:** 10.1186/s12913-025-13088-8

**Published:** 2025-07-12

**Authors:** Mathieu JP Poirier, Tina Nanyangwe-Moyo, Natalia Pires de Vasconcelos, Daniel Wang, Gigi O Lin, Ana Luiza Chieffi, Cauê Freitas Monaco, Zun Ge Mao, Steven J Hoffman

**Affiliations:** 1 Global Strategy Lab, Dahdaleh Institute for Global Health Research, Faculty of Health, York University, 4700 Keele Street, Dahdaleh Building 2120, Toronto, ON M3J 1P3 Canada; 2 School of Global Health, York University, Toronto, Canada; 3 Department of Sociology, University of Georgia, Athens, USA; 4 Fundação Getulio Vargas, Law School in São Paulo, Brazil; 5 Department of Health of the State of São Paulo, São Paulo, Brazil; 6 Centro Universitário São Camilo, School of Medicine, São Paulo, Brazil; 7 Osgoode Hall Law School, York University, Toronto, Canada

**Keywords:** Health technology assessments, Brazil, Court decisions, Treatments, CONITEC, Universal health coverage

## Abstract

**Background:**

The use of health rights litigation as a parallel decision-making venue to bypass health technology assessments (HTA) has resulted in unintended inequitable impacts on Latin American health systems since the 1990s. Brazil created a new HTA body in 2011 to promote a transparent and evidence-informed process in the Ministry of Health´s decisions about treatment coverage, but its impact on the judicial system’s provision of specific treatments to patient litigants has not yet been quantitatively evaluated.

**Methods:**

We leverage a unique dataset of 3,774 judicial claims for ten of Brazil’s most frequently litigated treatments to conduct the first quasi-experimental evaluation of treatment-specific changes in judicial decisions providing treatments through the national health system. Interrupted time series analyses using ordinary least-squares and logistic fractional response regressions were used to determine if an HTA recommendation was significantly associated with a change in court decisions in favour of litigants following a positive or negative recommendation for national coverage for the ten most litigated treatments in the country.

**Results:**

We find no evidence of a statistically significant change in court decisions using ordinary least-squares regression and decreases of smaller than 2.1% in positive court decisions using logistic fractional regression, regardless of whether HTA recommended for or against coverage. Among treatments recommended for coverage, three treatments experienced decreases in positive court decisions ranging from 3.1 to 26.8%. Among treatments recommended against coverage, two treatments experienced decreases in positive decisions ranging from 5.2 to 14.2%, and one treatment experienced a 9.6% increase in positive decisions.

**Conclusions:**

Our results demonstrate that nearly all court claims filed for the ten most litigated treatments in Brazil were granted, and HTA recommendations had almost no impact on judicial decisions to grant patient petitions for coverage. Policymakers should be aware that the creation of an HTA does not guarantee that its recommendations will produce a change in court decision making on patient petitions for treatment coverage. To realize the promise of basing difficult decisions on the provision and allocation of health technologies on principles of clinical utility, cost-effectiveness, and equity, the failure to meaningfully incorporate HTA in judicial processes must be addressed.

**Supplementary Information:**

The online version contains supplementary material available at 10.1186/s12913-025-13088-8.

## Introduction

Health technology assessments (HTAs) have been recognized by agencies such as the World Health Organization as an invaluable technical innovation for promoting evidence-based decision-making and maximizing value for money in health systems [1]. As appraisal frameworks, HTAs produce rigorous reviews of medical technologies, typically evaluating their medical and social value through a cost-effectiveness framework to determine whether to allow the use and coverage of medical devices, procedures, drugs, and treatments [2]. These considerations not only take into account clinical utility, but also weigh cost-effectiveness and other value-based considerations such as equity against the health system’s fiscal limitations [3].

While HTAs are now widely accepted and increasingly adopted in low- and middle-income countries (LMICs) like Thailand, Kenya, and Zambia [4], evidence of their utilisation by relevant actors– including policymakers, health practitioners, legal experts, and manufacturers– has largely focused on high-income countries (HICs) [1, 5, 6]. In LMICs where HTAs guide priority setting with far greater fiscal limitations, the process of institutionalization faces several challenges. These include a lack of awareness by the public and policymakers of the purpose and promise of HTAs [7], inadequacy of resources and technical expertise available to support HTAs, and systemic fragmentation in decision-making processes which affect healthcare services delivery [8].

In some countries with a constitutional right to healthcare [9], patients unable to access treatments through public healthcare systems can challenge the denial through judicial systems to rectify the potential infringement of their constitutional rights. As a result, those with the resources to afford legal services and the literacy and access to navigate the legal system have launched lawsuits against healthcare systems in countries where these constitutional provisions exist [10, 11]. Courts must then resolve tensions between individuals’ appeals for their constitutional rights to healthcare and policy decisions made by healthcare authorities, often through evidence-based regulatory guidance produced by HTAs.

Thanks to tens of thousands of court cases that are filed each year to obtain access to treatments, Brazil is an ideal context in which to evaluate the real-world impact of HTA on litigation for access to health technologies. The 1988 Brazilian constitution provided a comprehensive right to health that has entitled citizens to sue the state when seeking funding for treatments that the public national healthcare system (*Sistema Único de Saúde*– SUS) has denied [12]. Claimants’ high rates of success, combined with better access to judicial services and the underfunding of SUS, led to massive health litigation in the country. Judicial decisions in favor of claimants were seen to not only disregard resource limitations but also the available scientific evidence, often mandating SUS to provide experimental and unsafe treatments [11, 13].

As a response to this massive volume of litigation, Law 12,401 was passed in 2011 creating CONITEC (*Comissão Nacional de Incorporação de Tecnologias no Sistema Único de Saúde*), a technical commission responsible forHTA that should inform the Ministry of Health´s decisions about treatment coverage. Legislators and policymakers expected that a more transparent and scientifically robust decision-making process at CONITEC would lead to more deference from courts when they examine the health system´s priority-setting decisions [13]. However, litigation has allowed patients to circumvent HTA by claiming access to treatments that have not been assessed– or even recommended against– by CONITEC.

In a previous study [12], we found that the creation of CONITEC did not change courts’ behavior, with courts deciding in favor of patients in most cases, whether or not a treatment was recommended for coverage. However, it is possible that this overall result could obscure significant treatment-specific impacts if– whether due to cost, salience, or disease severity– courts are influenced by HTA recommendations for some treatments more than others. Here we leverage a unique dataset of thousands of judicial claims to conduct interrupted time series analyses (ITS) for the ten most frequently litigated treatments in Brazil, exploring the effect of CONITEC recommendations on judicial decisions in favour of funding treatments through the national health system.

## Methods

### Data

Our team compiled a random sample of all cases filed in state and federal courts against Brazil’s public health system covering the first 52 months of CONITEC’s creation from January 2011 to April 2015 in the capital cities of three Brazilian states: São Paulo, São Paulo; Porto Alegre, Rio Grande do Sul; and Florianópolis, Santa Catarina. These were chosen to maximize the chances of finding a significant impact under the assumption that public attorneys and staff specialized in responding to claims for healthcare treatments in these large capital cities are best prepared to defend the national health system in court [14]. Given the large number of cases, a simple random sampling technique was used for each state-year-court combination. The sample size for each combination was chosen to achieve a margin of error of ± 4% at 95% confidence level within each state-year. We excluded cases that were protected by a court order, involved a class action with indeterminate claimants, or if the court files were missing either the claimants’ or respondents’ briefs or the judgments. Detailed data collection and verification procedures detailed by Wang et al. [12] produced a final dataset containing 5,831 petitions for treatments that received 13,263 judicial decisions through the appeal process from preliminary and first instance decisions to second instance courts, the Superior Court of Justice (STJ), and the Supreme Federal Court (STF).

Court files of each claim were manually reviewed in each city’s public attorneys’ office using a codebook to extract information on the claimant’s illness, the health treatment requested by the claimant, whether the treatment had marketing authorization by Brazil’s medical products regulatory agency (*Agência Nacional de Vigilância Sanitária* - ANVISA), whether it was covered by the national health system (SUS), and whether a CONITEC assessment was cited by litigants. A pharmacist and a medical doctor then reviewed these claims to standardize treatment names, determine whether and when the claimed treatment was assessed by CONITEC, and whether the patient’s illness matched the illness for which CONITEC assessed the treatment (Table S1). Using this dataset, this study purposively selected the ten most commonly litigated treatments in this dataset, resulting in 3,774 judicial decisions, in order to find specific treatment effects that could be different from the overall result. In order of frequency, these treatments included analogous insulin (*N* = 1,350), hepatitis C virus (HCV) protease inhibitors (*N* = 471), ranibizumab (*N* = 369), tiotropium (*N* = 338), antipsychotics for bipolar disorder (*N* = 275), trastuzumab (*N* = 254), temozolomide (*N* = 206), new antivirals for Hepatitis C (*N* = 186), enoxaparin (*N* = 170), and hormone therapy for prostate cancer (*N* = 155).

### Statistical analysis

Our initial analyses used ITS estimated by ordinary least-squares (OLS) regression with Newey-West standard errors to test whether there was a change in the weighted monthly proportion of court decisions granting coverage to patients after a CONITEC recommendation was issued for that treatment [15]. Since positive and negative CONITEC recommendations cannot be randomly assigned to different court cases, this quasi-experimental method leverages the assumption that in the absence of a consequential intervention, the proportion of court cases granting access to the treatment should remain constant over time. On the other hand, if there is a statistically significant change in either the level (i.e. a one-time jump) or the trend (i.e. a gradual change over time) of the proportion of positive court decisions starting the month after a CONITEC recommendation, the null hypothesis would be rejected. Our model assumes that the impact of a CONITEC recommendation will begin to take effect within a month, and can be represented as Y_t_ = β_0_ + β_1_T + β_2_X_t_ + β_3_TX_t_, where Y_t_ is the proportion of positive court decisions in a given month, T is the time elapsed, X_t_ is a binary variable denoting the intervention point, β_0_ is the baseline level, β_1_ is the underlying time trend, β_2_ is a one-time level change at the time of intervention, and β_3_ is a slope change at the time of intervention [16]. Our primary analysis jointly evaluated all treatments that received a positive CONITEC recommendation and separately evaluated all treatments that received a negative CONITEC recommendation by aligning the month of recommendation for each treatment as the single intervention date. After restricting inclusion to decisions falling within 30 months before or after the intervention, a maximum of 3,251 decisions over a span of five years were included in analysis. A 30-month analysis window was selected to ensure a maximize the number of balanced pre- and post-intervention observations for conducting ITS analysis [17]. Monthly positivity rates were weighted by the number of court rulings per month and evaluated for autocorrelation and stationarity using augmented Dickey-Fuller tests (Table S2) [15]. We separately evaluated the effect of a CONITEC recommendation for the ten most commonly litigated treatments, of which five treatments received a positive CONITEC recommendation (for SUS coverage) and five received a negative CONITEC recommendation (against SUS coverage).

However, with monthly positivity rates consistently exceeding 90%, month-to-month variation in treatment-specific analyses using OLS-based ITS (Table S3) produced unrealistic counterfactual trends in excess of 100%. To address this challenge, we employed segmented logistic fractional response regressions with robust standard errors, which allowed predicted values to vary within a restricted range of 0 to 100% [18]. We then conducted a pairwise comparison of margins in the month immediately following CONITEC recommendation and in the final month of the analysis period. Similar to the standard OLS-based ITS approach, these estimates represent the absolute change in observed monthly positivity rate as compared to the pre-intervention time trend, while keeping counterfactual trends under the theoretical maximum of 100%.

All treatment-specific analyses were restricted to the maximum possible symmetric pre- and post-intervention times to mitigate the impact of the varying number of months before and after a CONITEC decision for each treatment. Robustness checks were run to account for the uncertain lag times between a CONITEC decision and a change in litigation outcomes, with all primary analyses run separately for a total sample period of three, four, and five years. This strategy resulted in equal numbers of observations before and after each intervention point, with a range of between 36 months (three years) and 60 months (five years) of data for joint analyses of all treatments that received a positive or negative CONITEC recommendation, and a range of between 18 months (HCV protease inhibitors) and 60 months (enoxaparin) of data for treatment-specific analyses.

## Results

**Fig. 1:**
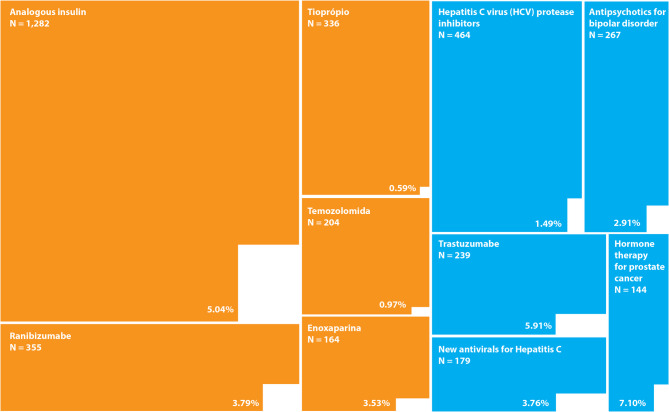
Proportion of negative and positive court outcomes. Tree map of court claim distribution. Negative (orange) or positive (blue) CONITEC recommendations are presented with treatment name, number of court claims, and the relative proportion of positive (blue or orange) and negative (white) judicial outcomes. The total number of court decisions (N) and the percentage of negative court outcomes are detailed for each treatment

There was very little variation in the high proportion of positive court rulings, regardless of whether CONITEC recommended for (1,293 of 1,341 decisions in favour of the patient) or against (2,341 of 2,433 decisions in favour of the patient) treatment coverage. The full distribution of petitions for treatments and proportion of positive court decisions is visualized in Fig. 1, demonstrating that courts decided in favour of patients between 92.9% (Hormone therapy for prostate cancer) and 99.6% (Tiotropium) of the time. This means that despite three of the top four most commonly litigated drugs of analogous insulin (35.8%), Ranibizumab (9.8%), and Tiotropium (9.0%) receiving a negative CONITEC recommendation, patient petitions were nearly guaranteed a positive court ruling in all cases. The second most commonly litigated drug and the only drug in this group with a positive recommendation was HCV protease inhibitors (12.5%).

OLS-based ITS analyses produced very little evidence of any change in court decisions after the publication of a CONITEC report, repeatedly finding no level or trend change in the proportion of decisions in favour of patients (Table 1; Fig. S1). No matter whether treatments were recommended for or against national health coverage, or whether outcomes were evaluated over a period of three, four, or five years, there was no significant change in the level or trend of decisions in favour of patients after an HTA was published.

**Table 1:** Time-aligned ITS analyses using ordinary least squares regression. The change in level and slope after the month of CONITEC evaluation for treatments receiving a positive (top) and negative (bottom) CONITEC recommendation over the span of three, four, and five years

	Treatments receiving a positive CONITEC recommendation		
Time interval	3 years	4 years	5 years
**Time trend** **(CI)**	0.001 (−0.002-0.004)	0.000 (−0.003-0.002)	0.000 (−0.002-0.002)
**Level change** **(CI)**	−0.019 (−0.066-0.027)	−0.009 (−0.046-0.029)	−0.015 (−0.050-0.020)
**Trend change** **(CI)**	0.001 (−0.004-0.006)	0.001 (−0.002-0.004)	0.001 (−0.001-0.004)
**Constant** **(CI)**	0.954** (0.916-0.992)	0.968** (0.929-1.006)	0.964** (0.926-1.001)
**Number of claims**	971	1106	1213

	Treatments receiving a negative CONITEC recommendation		
Time interval	3 years	4 years	5 years
**Time trend** **(CI)**	0.000 (−0.003-0.003)	0.001 (−0.001-0.003)	0.002* (0.000-0.003)
**Level change** **(CI)**	−0.011 (−0.048-0.025)	−0.012 (−0.043-0.019)	−0.012 (−0.042-0.019)
**Trend change** **(CI)**	0.002 (−0.001-0.005)	0.001 (−0.002-0.003)	−0.001 (−0.003-0.001)
**Constant** **(CI)**	0.960** (0.928-0.992)	0.943** (0.917-0.969)	0.925** (0.894-0.955)
**Number of claims**	1480	1784	2038

**p < 0.01, *p < 0.05; confidence intervals in parentheses

**Fig. 2:**
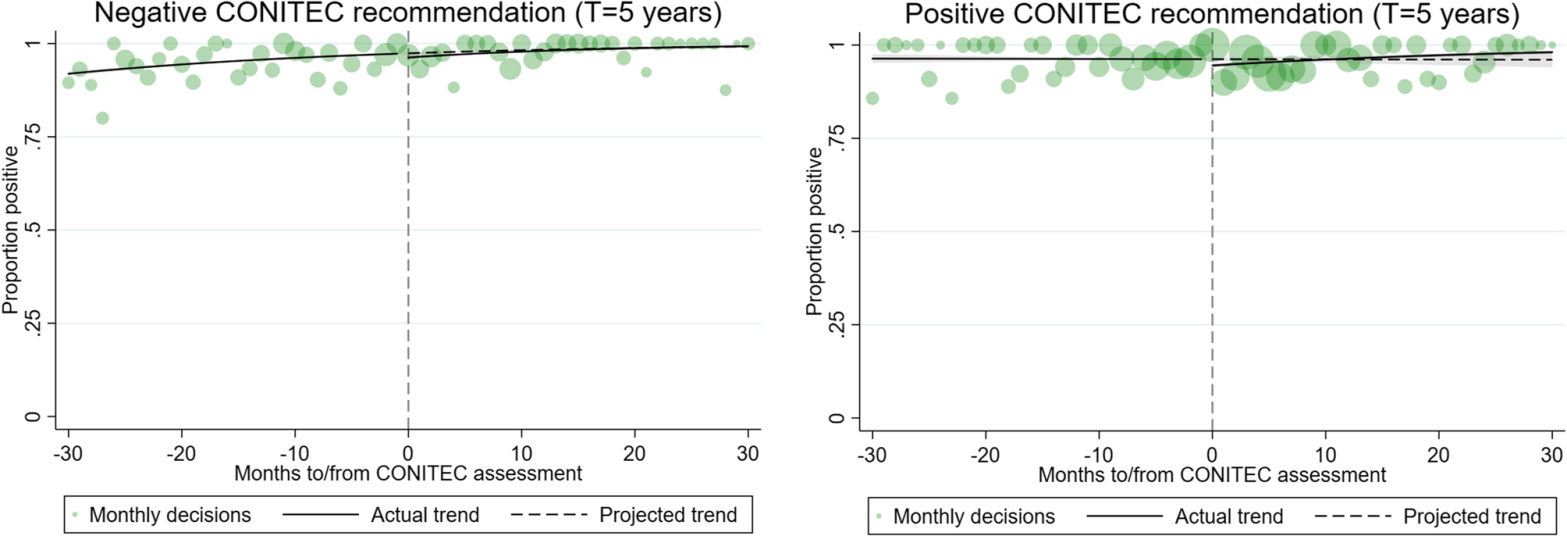
Interrupted time-series analyses using fractional logistic regression. ITS results for all treatments receiving a positive CONITEC recommendation (right) and all treatments receiving a negative CONITEC recommendation (left) over a total time period of five years with aligned CONITEC assessment dates. Monthly judicial positivity rates are presented in green with marker sizes adjusted to the number of court decisions in that month. Pre-intervention judicial outcomes are used to generate a projected trend over the following 30 months after the intervention (dashed line), which is then evaluated for significant differences with actual post-intervention judicial outcomes (solid line) at the point of intervention and 30 months after the intervention

Fractional regression models that account for data bounding similarly demonstrate almost no significant changes after CONITEC recommendations (Fig. 2). For treatments that received a positive CONITEC recommendation, there was a small decrease in the proportion of positive decisions ranging from 0.89 to 2.05% in the month following the recommendation, depending on which model was used (Table 2). However, by the final month of analysis, there was no significant difference between observed and expected proportions in all models. For treatments that received a negative CONITEC recommendation, there was also a small decrease in the proportion of positive decisions ranging from 1.11 to 1.80% in the month following the recommendation. By the final month of analysis, that decrease had either reversed to an increase of 2.06% for a three-year model, an increase of 0.83% for a four-year model, or levelled to no significant difference between observed and expected proportions in the five-year model.

**Table 2:** Interrupted time-series analyses using fractional logistic regression. The absolute difference and confidence intervals in monthly percentage of positive decisions between the actual and projected trend after the month of CONITEC evaluation and at the end of the analysis window for treatments receiving a positive (top) and negative (bottom) CONITEC recommendation over the span of three, four, and five years

	Treatments receiving a positive CONITEC recommendation		
	3 years	4 years	5 years
**Month of decision** **(CI)**	−2.05* (−2.86-1.25)	−0.89* (−1.61-0.17)	−1.70* (−2.40-1.01)
**End of analysis** **(CI)**	−0.06 (−1.34-1.22)	1.36 (−1.42-4.13)	2.02 (−0.09-4.12)
**Number of claims**	971	1106	1213

	**Treatments receiving a negative CONITEC recommendation**		
	**3 years**	**4 years**	**5 years**
**Month of decision** **(CI)**	−1.80* (−2.39-1.22)	−1.61* (−2.15-1.07)	−1.11* (−1.69-0.53)
**End of analysis** **(CI)**	2.06* (1.14-2.99)	0.83* (0.41-1.26)	0.11 (−0.28-0.50)
**Number of claims**	1480	1784	2038

*Statistically significant difference at the 95% confidence level

**Fig. 3:**
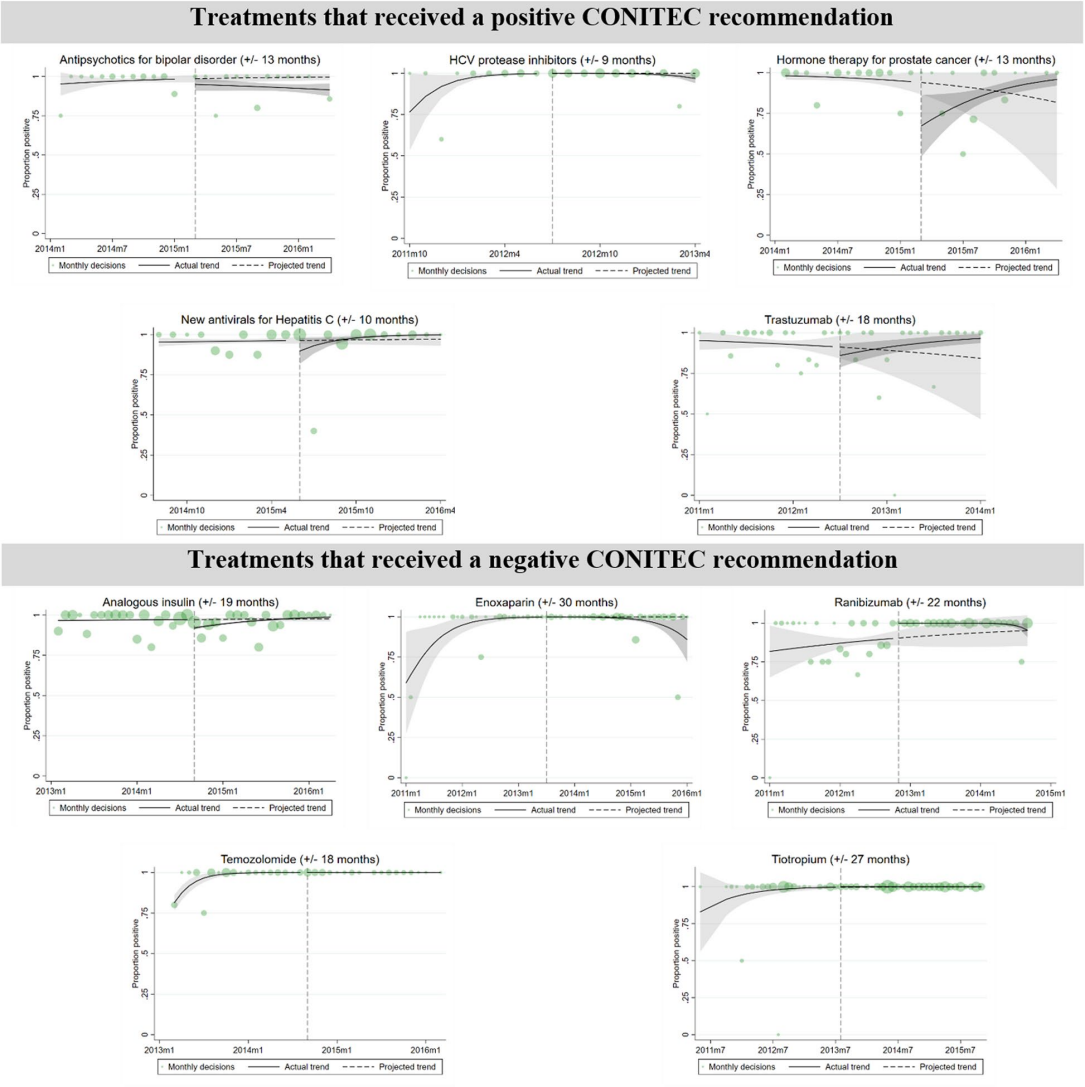
Treatment-specific interrupted time-series analyses using fractional logistic regression. Analyses for treatments that received a positive CONITEC recommendation (top) and treatments that received a negative CONITEC recommendation (bottom) with the number of months before and after CONITEC assessment noted for each treatment. Monthly positivity rates are presented in green with marker sizes adjusted to the number of court decisions in that month. Pre-intervention judicial outcomes are used to generate a projected trend after the intervention (dashed line), which is then evaluated for significant differences with actual post-intervention judicial outcomes (solid line) at the point of intervention and the final month included in analysis after the intervention

Treatment-specific analyses produced mixed results (Fig. 3), with decreases in positive court decisions occurring more frequently than increases following both positive and negative CONITEC assessments. For treatments receiving a positive CONITEC recommendation (Table S4.1), three treatments experienced a significant change, and both were significant decreases in positive decisions. There was no significant change in positive court decisions for Hepatitis C virus protease inhibitors in the month following the CONITEC decision, but a significant 3.13% decrease (CI −5.92% to −0.35%) was found by nine months after the decision. For hormone therapy for prostate cancer and antipsychotics for bipolar disorder, there were significant 26.75% and 8.09% decreases (CI −47.14% to −6.36%; −12.60% to −3.58%) in the month following the CONITEC decision, but these temporary declines reversed to no significant difference by 13 and 26 months after the decisions. No significant change was found for new antivirals for Hepatitis C or Trastuzumab.

Among treatments receiving a negative CONITEC recommendation (Table S4.2), three treatments experienced a significant change, with a mixture of two decreases and one increase in positive decisions. For analogous insulin, there was a significant 5.23% decrease (CI −6.40% to −4.07%) in the month following the CONITEC decision, but this temporary decline reversed to no significant difference by 19 months after the decision. There was no significant change in positive court decisions for Enoxaparin in the month following the CONITEC decision, but a significant − 14.20% decrease (CI −28.00% to −0.40%) was found by 30 months after the decision. Finally, there was a significant 9.59% increase (CI 4.64–14.53%) in the month following the CONITEC decision for Ranibizumab, but this reversed to no significant difference by 22 months after the decision. No significant change was found for Temozolomide or Tiotropium.

## Discussion

Using rigorous quasi-experimental methods to evaluate a novel dataset of thousands of patient claims to government coverage for treatment, we find that HTAs conducted by CONITEC produced little to no impact on the ten most commonly litigated treatments in Brazil. For treatments with a positive CONITEC assessment which would be expected to increase the odds of subsequent positive court decisions, we instead found that positive court decisions for hormone therapy for prostate cancer and antipsychotics for bipolar disorder temporarily decreased in the month after assessment, and decisions for Hepatitis C virus protease inhibitors decreased by the end of the analysis period. For treatments with a negative CONITEC assessment which would be expected to decrease the odds of subsequent positive court decisions, positive court decisions for analogous insulin temporarily decreased in the month after assessment and decisions for Enoxaparin decreased by the end of the analysis period. At the same time, favourable rulings for Ranizumab– a treatment not recommended for SUS coverage– increased in the month after CONITEC assessment.

Our time-aligned grouped analysis found no significant change in level or slope of positive court decisions over a span of three, four, or five years using OLS-based ITS analysis for both treatments receiving a positive recommendation as well as those receiving a negative recommendation. Fractional-regression-based ITS analysis similarly identifies small decreases of up to 2.1% in the month after CONITEC recommendation, which quickly attenuated with courts granting access to patients in over 95% of cases. While no significant change was found at the end of three, four, or five years for treatments receiving a positive recommendation, those receiving a negative recommendation experienced a small 2% increase at the end of the three-year analysis window, although this eventually dissipated to no significant change using a five-year analysis window.

### Economic social, and health consequences

 Our findings corroborate evidence from previous studies which have found judicial rulings in Brazilian courts to favour litigants irrespective of CONITEC recommendations [12, 19]. The cumulative impact of this bias can have significant financial implications for public healthcare systems without improvements to public health. For example, a recent clinical study found no significant difference in treatment effectiveness for macular degeneration between Ranibizumab and Bevacizumab, with Ranibizumab requiring more than R$2 million (Brazilian Reais) to generate an additional quality-adjusted life year for patients [20]. There is also no robust evidence that the use of analogous insulin in usual practice provide better clinical outcomes compared to NPH insulin [21]. Yet, according to the CONITEC reports that did not recommend the coverage of analogous insulin in 2014, the budget impact of these drugs on the health system would have been around R$16 billion (around 3.2 billion USD) over a period of five years [22]. Nevertheless, courts have been found to weigh physicians’ prescriptions and claims to constitutional privilege with the same or greater weight than systematic priority-setting evidence by HTAs [12].

 The lack of responsiveness of courts to HTAs in Brazil has two important economic and public health consequences. First, the near guarantee of court-mandated treatment coverage for those able to afford access to the judicial system can aggravate inequities in access to healthcare by skewing public health spending in favour of those with the ability to access litigation rather than population health needs [23– 25]. For example, between January 2010 and July 2017, the Brazilian Ministry of Health purchased 812 drugs to comply with court orders, costing the system an additional R$5.5 billion [26]. Second, facilitating access to treatment that did not go through a robust HTA can lower the level of care and even lead to adverse patient outcomes. Over the same time period, a sample of over 40 commonly litigated drugs worth R$3.2 billion (approximately US$600 million) of public health spending were associated with 1,248 serious adverse events [26].

The most straightforward explanation for the underutilisation of HTA by courts in Brazil is insufficient awareness of CONITEC recommendations and lack of understanding of the HTA process [10]. We have previously demonstrated that CONITEC recommendations were not cited in 59.4% of court cases, despite 96.5% of these cases being granted a positive verdict [12]. Around the same time CONITEC was created, health law and SUS regulation were not required subjects in the public entrance exams of judges, leading the agency within the judicial system responsible for regulating courts’ administrative and financial activities (the National Council of Justice), to recommend the addition of these subjects for all such exams in the country [11]. Lack of health law awareness is potentially worse in state courts, where most health litigation occurs because federal judges are more likely to have experience and better training in administrative law that includes health law and SUS regulation. In a previous study, we found evidence in favor of that hypothesis– when compared to state courts, federal courts were less likely to grant health litigation claims even after we controlled for the existence of a CONITEC recommendation in a particular case [12].

The structure of the judicial process in individual claims might also explain the phenomenon, as it reduces issues of policy and distributive justice to a conflict between the individual versus the government and creates the identified-victim effect, making it likely that decision-makers will follow the rule of rescue to benefit an individual at the expense of statistical lives [27]. Another potential explanation is the country’s civil law structure in which precedents are rarely binding and do not establish *stare decisis.* Therefore, judges may see their decisions as isolated exceptions for individual patients, while overlooking the broad policy implications that thousands of such rulings can have on the healthcare system [13]. Additionally, the existence of judicial activism in Brazil (a phenomenon where courts render decisions for a secondary purpose of catalyzing social change or new judicial debate) can complicate HTA-uptake [28], especially considering how current trends in health litigation in the country have been traced back to 1990s’ changes in legal culture among judges about the enforceability of constitutional rights, and especially social and economic rights [23].

### Implications for policy

Across Latin America, legal claims for medicines have varied depending on the structure, coverage and quality of health systems and the level of judicial enforcement of the right to healthcare. The literature about the issue in the region is vast and multidisciplinary, offering multiple avenues for description and interpretation. For instance, in Chile, for example, recent scholarship has found that litigation for treatments is less common compared to Brazil, Colombia, and Argentina [10]. This may be due to an interpretation of the right to health in the 1980 Constitution making it difficult for individuals to file lawsuits for treatments, as well as a delay in publishing the list of treatments covered under the universal healthcare plan until 2005 [29]. In the case of Peru and Colombia, despite significant expansion in health insurance coverage, recent work has argued that inequalities have provoked grievances for access to treatments since the mid-1990s [30]. Therefore, strategic litigation have served as an alternative to gain access to healthcare coverage for specific treatments and to advocate for progress towards the realization of the right to healthcare. However, in other LMICs with institutionalized HTAs, such as Indonesia and Thailand, lawsuits regarding coverage decisions are not commonplace, but HTA evidence remains underused due to lack of coordination between the HTA process and key decision makers [31, 32].

In the case of Brazil, the Supreme Federal Court (STF) is yet to make a binding decision in the case RE 566,471 to determine in which circumstances courts can order the public health system to provide non-listed treatment. Some Justices propose that this should only be allowed when the treatment is yet to be assessed by CONITEC, but not if it has been assessed and coverage was not recommended. Others are concerned that this approach may reduce access to treatments and unduly restrict the right to health, leading to a stalemate in the STF on this issue. In the absence of a binding Supreme Court decision, technical teams have been created to assist lower courts’ decision-making and verification of evidence [9]. However, courts cannot be compelled to consult these technical teams and questions can be raised regarding the quality of the assessment and the fact that they rarely consider cost-effectiveness analysis and budget impact [10].

In the future, Brazil could look to programs such as one implemented by the Ministry of Health in Uruguay to actively educate defense attorneys and judges on the interpretation and utilisation of evidence generated by HTAs. This initiative has effectively helped to reduce the number of court warrants for mandatory treatment and access treatments by at least 25%, offering one promising strategy to incentivize the buy-in of legal systems in the region [28]. Partnerships between health departments and defense attorneys for pre-trial settlements are already a reality in many Brazilian states [14], but they could be expanded into more a comprehensive training about the different ways claimants can have their health needs met within the current health policy or contest its choices through the HTA system by helping patients and lawyers act at the regulatory level to have new technologies evaluated and potentially incorporated by CONITEC. Another important source of support can come from international networks such as Health Technology Assessment International (HTAi) and the International Network of Agencies for Health Technology Assessment (INAHTA) that have previously enhanced national capacity to execute HTA evaluations through capacity building programmes [3, 29].

### Strengths and limitations

This is the first quasi-experimental study to evaluate judicial deference to evidence generated through HTA for specific medical treatments. Our analysis of 3,774 judicial decisions on the ten most litigated treatments in the Brazilian legal system allows us to generate evidence for the treatments that are most responsible for driving national health system spending. Since HTAs aim to rationalize decision-making on which treatments are best able to produce population health impact with limited budgets, this allowed us to probe policy-relevant questions of whether these priority-setting mechanisms are being undermined by a lack of adherence in legal systems. The use of both OLS- and logistic fractional response-based ITS analyses, in addition to sensitivity checks using three-, four-, and five-year models strengthen our confidence in the findings.

Despite these strengths, our data are limited to judicial claims in three major Brazilian states, which may not fully capture regional dynamics across the country. Our analysis is also limited to the first 52 months following CONITEC’s establishment, potentially limiting insights into longer-term trends or more recent policy adaptations. The use of fractional response regression models is a novel way to address issues related to boundedness, but more research is needed to ascertain its sensitivity to different model specifications and small sample sizes. Lastly, our findings offer important insights into the challenges of institutionalizing evidence-informed HTA recommendations to inform judicial decisions for the Brazilian healthcare system, but their generalizability to other LMICs with different legal frameworks and healthcare systems may be limited.

## Conclusion

Implementing a new HTA process to base treatment coverage decision-making on principles of fairness and efficiency does not guarantee that its recommendations will perceived by all actors as legitimate decisions. We find little to no evidence that HTAs of the ten most commonly litigated treatments in Brazil had any impact on the judicial system’s near-universal tendency to grant access to patient claims. Unchecked litigation to access medical treatments not recommended for coverage by HTAs can lead to inequitable health system spending and adverse health impacts. Our findings quantify an entrenched disconnection between decisions made by the national HTA agency of Brazil and judicial rulings to grant access to drugs to individual litigants. To realize the promise of basing difficult decisions on the provision of health technologies on principles of clinical utility, cost-effectiveness, and equity, the failure to meaningfully incorporate HTA in judicial processes must be addressed. The experience of Brazil should caution against assumptions that HTAs in Latin America and LMICs are inexorably taken into consideration by judicial systems. Instead, a concerted effort must be made to educate and equip courts with the ability to incorporate HTA into their decision-making processes.

## Supplementary Information

Supplementary Material 1.

## Data Availability

The full open-access dataset is available at: Wang, Daniel (2020), “Data for: Health Technology Assessment and Judicial Deference to Priority-Setting Decisions in Health Care: Evidence from Brazil”, Mendeley Data, V1, 10.17632/vvhk5pm7m2.1.

## Abbreviations

ANVISA: Agência Nacional de Vigilância Sanitária
CONITEC: Comissão Nacional de Incorporação de Tecnologias no Sistema Único de Saúde
HICs: High-income countries
HTA: Health technology assessment
LMICs: Low- and middle-income countries
SUS: Sistema Único de Saúde
