# Supplementary material for: Treatment-specific interrupted time series analyses of judicial deference to health technology assessment in Brazil
Source: BMC Health Serv Res. 2025 Jul 12;25:950. doi: 10.1186/s12913-025-13088-8 (PMC12254987; doi:10.1186/s12913-025-13088-8)
Supplement: Supplementary file 1 — Supplementary Material 1. [file 12913_2025_13088_MOESM1_ESM.docx]

Supplementary Material

**Treatment-Specific Interrupted Time Series Analyses of Judicial Deference to Health Technology Assessment in Brazil**

Mathieu JP Poirier^1,2*^, Tina Nanyangwe-Moyo^1^, Natalia Pires de Vasconcelos^3^, Daniel Wang^4^, Gigi O Lin^1^, Ana Luiza Chieffi^5^, Cauê Freitas Mônaco^6^, Michelle Mao^1^, Steven J Hoffman^1,2,7^

^1^ Global Strategy Lab, Dahdaleh Institute for Global Health Research, Faculty of Health, York University, Toronto, Canada

^2^ School of Global Health, York University, Toronto, Canada

^3^ Department of Sociology, University of Georgia, Athens, United States

^4^ Fundação Getulio Vargas, Law School in São Paulo, Brazil

^5^ Deapartment of Health of the State of São Paulo, São Paulo, Brazil

^6^ Centro Universitário São Camilo, School of Medicine, São Paulo, Brazil

^7^ Osgoode Hall Law School, York University, Toronto, Canada

**Table S1. Characteristics of the ten most commonly litigated treatments in Brazil.**

| **Treatment** | **Treatment description** | **Date of CONITEC recommendation** | **SUS coverage?** |
| --- | --- | --- | --- |
| **Positive CONITEC recommendation** | | | |
| Antipsychotics for bipolar disorder | Clozapine, lamotrigine, quetiapine, olanzapine, and risperidone for the treatment of bipolar disorder (IC10 F31), | March 2015 | Yes, but for a different health condition |
| Hepatitis C virus (HCV) protease inhibitors | Boceprevir and telaprevir for the treatment of chronic viral hepatitis C (ICD10 B18.2). | July 2012 | No |
| Hormone therapy for prostate cancer | Hormone therapy in patients who will undergo radiotherapy for prostate cancer treatment (ICD C61). | March 2015 | No |
| New antivirals for Hepatitis C | Sofosbuvir, simeprevir, and daclatasvir for the treatment of chronic viral hepatitis C (ICD10 B18.2). | June 2015 | No |
| Trastuzumab | Tratuzumab for the treatment of breast cancer (ICD C50) after surgery, chemotherapy, and radiotherapy. | July 2012 | No |
| **Negative CONITEC recommendation** | | | |
| Analogous insulin | Long-acting insulin analogues (detemir and glargine) and short- acting insulin analogues  (lispro, aspart, and glulisine) for the treatment of diabetes mellitus types 1 (ICD E10) and 2 (ICD E11). | Sept 2014 | No |
| Enoxaparin | Enoxaparin for the treatment of thrombophilia in pregnant and puerperal women (ICD I82) | July 2013 | No |
| Ranibizumab | Ranibizumab for the treatment of age-related macular degeneration (AMD) (ICD H35.31 or H35.32) | Nov 2012 | No |
| Temozolomide | Temozolamide as an adjunct treatment to surgery and radiotherapy for patients with high-grade gliomas (C71) | Sept 2014 | No |
| Tiotropium | Tiotropium bromide for the treatment of chronic obstructive pulmonary disease (ICD J44) | Aug 2013 | Yes in 5 states for high-risk patients |

Details of treatment name, treatment description, the month of the CONITEC assessment, and whether the treatment was already available through SUS coverage.^1^

**Table S2. Dickey-Fuller tests results.** Test statistics and P-values reported for each treatment evaluated.

| **Treatment name** | **Test statistic** | **P-value** |
| --- | --- | --- |
| Antipsychotics for bipolar disorder | -3.567 | 0.005 |
| Hepatitis C virus (HCV) protease inhibitors | -7.664 | 0.000 |
| Hormone therapy for prostate cancer | -6.428 | 0.000 |
| New antivirals for Hepatitis C | -4.911 | 0.000 |
| Trastuzumab | -6.746 | 0.000 |
| Analogous insulin | -7.511 | 0.000 |
| Enoxaparin | -8.328 | 0.000 |
| Ranibizumab | -13.264 | 0.000 |
| Temozolomide | -7.490 | 0.000 |
| Tiotropium | -7.329 | 0.000 |

**Table S3. Treatment-specific OLS-based ITS results.**

| **Table S3.1 Single group ITS** | **Treatments that received a positive CONITEC recommendation** | | | |
| --- | --- | --- | --- | --- |
| **Treatment name** | **Antipsychotics for bipolar disorder** | **Hepatitis C virus (HCV) protease inhibitors** | | **Hormone therapy for prostate cancer** |
| **Time trend (CI)** | 0.000  (-0.002 - 0.001) | 0.020  (-0.016 - 0.055) | | 0.000  (-0.003 - 0.004) |
| **Level change (CI)** | -0.029  (-0.138 - 0.081) | -0.055  (-0.144 - 0.033) | | -0.238*  (-0.448 - -0.028) |
| **Trend change (CI)** | -0.003  (-0.014 - 0.009) | -0.020  (-0.056 - 0.016) | | 0.021* (0.000 - 0.041) |
| **Constant (CI)** | 0.984 (0.940 - 1.028) | 0.867 (0.630 - 1.104) | | 0.946 (0.835 - 1.056) |
| **Number of claims** | 275 | 471 | | 155 |
|  | **New antivirals for Hepatitis C** | | **Trastuzumab** | |
| **Time trend (CI)** | -0.001  (-0.010 - 0.008) | | -0.002  (-0.014 - 0.010) | |
| **Level change (CI)** | -0.038  (-0.228 - 0.152) | | -0.028  (-0.180 - 0.124) | |
| **Trend change (CI)** | 0.013  (-0.018 - 0.044) | | 0.006  (-0.007 - 0.018) | |
| **Constant (CI)** | 0.969 (0.907 - 1.031) | | 0.954 (0.834 - 1.074) | |
| **Number of claims** | 186 | | 254 | |

** p < 0.01, * p < 0.05; confidence intervals in parentheses

| **Table S3.2 Single group ITS** | **Treatments that received a negative CONITEC recommendation** | | | |
| --- | --- | --- | --- | --- |
| **Treatment name** | **Analogous insulin** | **Enoxaparin** | | **Ranibizumab** |
| **Time trend (CI)** | 0.002 (0.000 - 0.003) | 0.011 (0.000 - 0.022) | | 0.004  (-0.008 - 0.016) |
| **Level change (CI)** | -0.056*  (-0.104 - -0.009) | -0.061  (-0.179 - 0.058) | | 0.102* (0.003 - 0.201) |
| **Trend change (CI)** | 0.002  (-0.001 - 0.005) | -0.013*  (-0.025 - -0.001) | | -0.005  (-0.017 - 0.007) |
| **Constant (CI)** | 0.906 (0.869 - 0.944) | 0.760 (0.526 - 0.995) | | 0.822 (0.640 - 1.004) |
| **Number of claims** | 1350 | 170 | | 369 |
|  | **Temozolomide** | | **Tiotropium** | |
| **Time trend (CI)** | 0.000  (-0.001 - 0.000) | | 0.003  (-0.003 - 0.009) | |
| **Level change (CI)** | 0.020  (-0.011 - 0.052) | | -0.015  (-0.059 - 0.030) | |
| **Trend change (CI)** | 0.000 (0.000 - 0.001) | | -0.003  (-0.009 - 0.003) | |
| **Constant (CI)** | 0.996 (0.984 - 1.008) | | 0.922 (0.796 - 1.048) | |
| **Number of claims** | 206 | | 338 | |

** p < 0.01, * p < 0.05; confidence intervals in parentheses

Estimates and confidence intervals (CIs) were generated using ordinary least squares (OLS) regression-based interrupted time series analysis.

**Figure S1. Interrupted time-series analyses using fractional logistic regression.**

| **Fig. S1.1 Treatments that received a positive CONITEC recommendation** | | |
| --- | --- | --- |
| 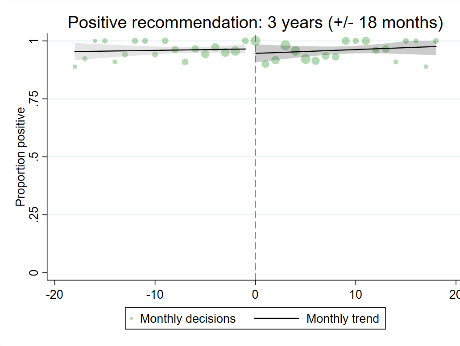 | 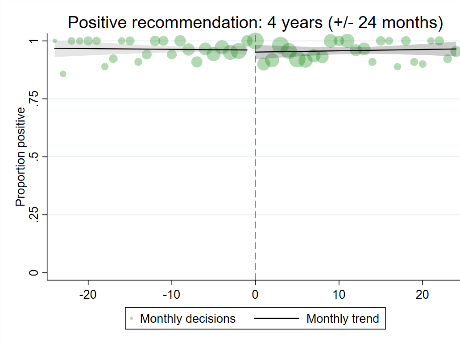 | 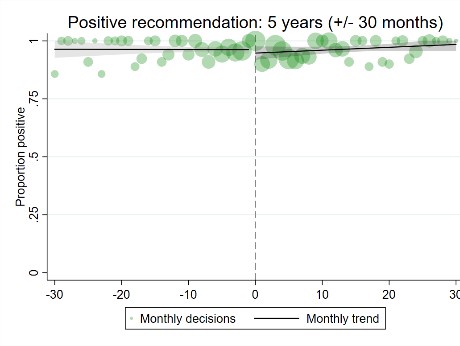 |
| **Fig. S1.2 Treatments that received a negative CONITEC recommendation** | | |
| 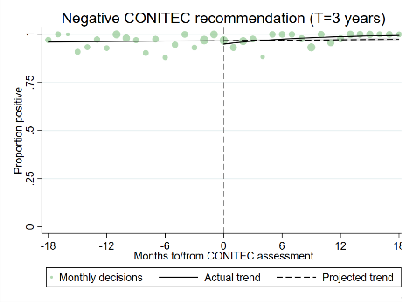 | 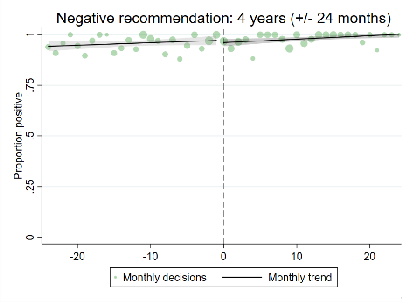 | 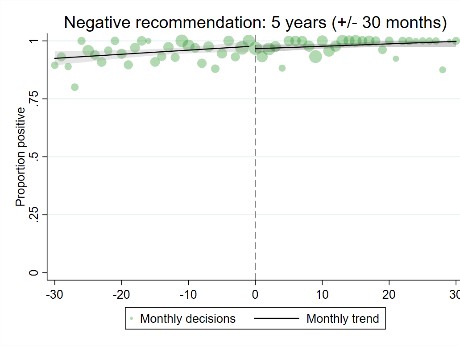 |

Analyses for treatments that received a positive CONITEC recommendation (top) and treatments that received a negative CONITEC recommendation (bottom) over the span of three, four, and five years. Monthly positivity rates are presented in green, with marker sizes adjusted to the number of court decisions in that month.

**Table S4. Treatment-specific interrupted time-series analyses using fractional logistic regression.**

| **Table S4.1** | **Treatments that received a positive CONITEC recommendation** | | | | | | | | | |
| --- | --- | --- | --- | --- | --- | --- | --- | --- | --- | --- |
|  | Antipsychotics for bipolar disorder | | Hepatitis C virus protease inhibitors | | Hormone therapy for prostate cancer | | New antivirals for Hepatitis C | | Trastuzumab | |
| Evaluation period | 26 months | | 18 months | | 26 months | | 20 months | | 36 months | |
| Evaluation point | Intervention | Endpoint | Intervention | Endpoint | Intervention | Endpoint | Intervention | Endpoint | Intervention | Endpoint |
| Months after decision | 0 | 13 | 0 | 9 | 0 | 13 | 0 | 10 | 0 | 18 |
| Percent difference | -3.67 | -8.09* | 0.09 | -3.13* | -26.75* | 14.12 | -6.58 | 2.71 | -5.15 | 12.29 |
| Lower CI | -8.30 | -12.60 | -0.05 | -5.92 | -47.14 | -39.25 | -14.88 | -1.33 | -15.30 | -25.27 |
| Upper CI | 0.95 | -3.58 | 0.23 | -0.35 | -6.36 | 67.49 | 1.71 | 6.74 | 4.99 | 49.84 |
|  |  |  |  |  |  |  |  |  |  |  |
| **Table S4.2** | **Treatments that received a negative CONITEC recommendation** | | | | | | | | | |
|  | Analogous insulin | | Enoxaparin | | Ranibizumab | | Temozolomide | | Tiotropium | |
| Evaluation period | 38 months | | 60 months | | 44 months | | 36 months | | 54 months | |
| Evaluation point | Intervention | Endpoint | Intervention | Endpoint | Intervention | Endpoint | Intervention | Endpoint | Intervention | Endpoint |
| Months after decision | 0 | 19 | 0 | 30 | 0 | 44 | 0 | 18 | 0 | 27 |
| Percent difference | -5.23* | 1.03 | 0.09 | -14.20* | 9.59* | 0.22 | 0.00 | 0.00 | 0.29 | 0.00 |
| Lower CI | -6.40 | -0.63 | -0.30 | -28.00 | 4.64 | -10.61 | -0.01 | 0.00 | -0.53 | -0.02 |
| Upper CI | -4.07 | 2.68 | 0.47 | -0.40 | 14.53 | 11.05 | 0.02 | 0.00 | 1.12 | 0.03 |

Pairwise comparison of margins between expected and actual positive court decisions for treatments that received a positive CONITEC recommendation (top) and treatments that received a negative CONITEC recommendation (bottom). Absolute differences in the percentage of positive court decisions with upper and lower confidence intervals are presented.

**References**

1. Ministério da Saúde. Recomendações da Conitec [Internet]. 2023 [cited 2023 Aug 7]. Available from: https://[www.gov.br/conitec/pt-br/assuntos/avaliacao-de-tecnologias-em-](http://www.gov.br/conitec/pt-br/assuntos/avaliacao-de-tecnologias-em-) saude/recomendacoes-da-conitec
